# Cytosolic chloride ion is a key factor in lysosomal acidification and function of autophagy in human gastric cancer cell

**DOI:** 10.1111/jcmm.12257

**Published:** 2014-04-12

**Authors:** Shigekuni Hosogi, Katsuyuki Kusuzaki, Toshio Inui, Xiangdong Wang, Yoshinori Marunaka

**Affiliations:** a Department of Molecular Cell Physiology, Graduate School of Medical Science, Kyoto Prefectural University of MedicineKyoto, Japan; b Japan Institute for Food Education and Health, Heian Jogakuin (St. Agnes') UniversityKyoto, Japan; c Department of Orthopaedic Surgery, Kyoto Kujo HospitalKyoto, Japan; d Department of Bio-Ionomics, Graduate School of Medical Science, Kyoto Prefectural University of MedicineKyoto, Japan; e Saisei Mirai ClinicsMoriguchi, Japan; f Department of Respiratory Medicine, Shanghai Respiratory Research Institute, Fudan University Zhongshan HospitalShanghai, China

**Keywords:** autophagy, chloride ion, lysosome, pH, apoptosis

## Abstract

The purpose of the present study was to clarify roles of cytosolic chloride ion (Cl^−^) in regulation of lysosomal acidification [intra-lysosomal pH (pH_lys_)] and autophagy function in human gastric cancer cell line (MKN28). The MKN28 cells cultured under a low Cl^−^ condition elevated pH_lys_ and reduced the intra-lysosomal Cl^−^ concentration ([Cl^−^]_lys_) *via* reduction of cytosolic Cl^−^ concentration ([Cl^−^]_c_), showing abnormal accumulation of LC3II and p62 participating in autophagy function (dysfunction of autophagy) accompanied by inhibition of cell proliferation *via* G_0_/G_1_ arrest without induction of apoptosis. We also studied effects of direct modification of H^+^ transport on lysosomal acidification and autophagy. Application of bafilomycin A1 (an inhibitor of V-type H^+^-ATPase) or ethyl isopropyl amiloride [EIPA; an inhibitor of Na^+^/H^+^ exchanger (NHE)] elevated pH_lys_ and decreased [Cl^−^]_lys_ associated with inhibition of cell proliferation *via* induction of G_0_/G_1_ arrest similar to the culture under a low Cl^−^ condition. However, unlike low Cl^−^ condition, application of the compound, bafilomycin A1 or EIPA, induced apoptosis associated with increases in caspase 3 and 9 without large reduction in [Cl^−^]_c_ compared with low Cl^−^ condition. These observations suggest that the lowered [Cl^−^]_c_ primarily causes dysfunction of autophagy without apoptosis *via* dysfunction of lysosome induced by disturbance of intra-lysosomal acidification. This is the first study showing that cytosolic Cl^−^ is a key factor of lysosome acidification and autophagy.

## Introduction

Autophagy has been found to be a self-nutrient-providing system for cell survival under starvation conditions [1–3], and an important system for degradation of miss-folded or long-lived proteins, and superfluous or damaged organelle such as mitochondria [4–6]. Starvation-caused poverty of nutrients induces autophagy for cell survival *via* autophagy-mediated recycling of nutrients contained in cells themselves [1]. Cells mainly produce amino acids *via* autophagy-mediated process by digesting their own proteins [1]. New proteins are synthesized from these amino acids provided by autophagy [1]. As mentioned above, autophagy is, in general, activated by starvation. However, it has been recently suggested that autophagy process functions even under conditions with rich nutrition [7], and that impairment or activation of autophagy closely relates to pathogenesis of diverse diseases including Parkinson disease [6], diabetes mellitus [8], inflammatory disease such as Crohn disease [9] and cancer [10].

As cancer cells survive under hypoxic and hypo-nutrient microenvironments, cancer cells elevate autophagy ability to use recyclable materials [10]. It has been clarified that impairment of autophagy system by knocking down Atg5 or Atg7 induces apoptosis of cancer cells, inhibiting cell growth [11–13]. Autophagy is a catabolic process degrading cell components mediated through lysosomal machineries. Lysosome is, therefore, a key organelle in autophagy degrading various compounds [3]. In fact, at the final phase of degradation of proteins in autophagy process, lysosomes fuse to autophagosomes followed by lysosomal enzyme-mediated digestion of proteins.

The digesting activity of lysosomal enzymes depends on intra-lysosomal acidity, which is primarily generated by V-type H^+^-ATPase (proton pump) co-operating with ClC-7, Cl^−^/H^+^ antiporter, which is assumed to participate in Cl^−^ movement 14; ClC-7 has 2Cl^−^/1H^+^ exchange stoichiometry [15]. The ClC-7 located on lysosome membrane would primarily behave as a Cl^−^ permeation pathway in lysosomal membrane [14]. Mutation of ClC-7 induces abnormal accumulation of proteins into intra-lysosomal meaning disturbance of lysosomal function [16]. It is also reported that inhibition of ClC-7 by siRNA impairs lysosomal acidification [14] and induces abnormal accumulation of proteins in lysosomes resulting in inhibition of cell proliferation [17]. The observations [14,16] suggest that Cl^−^ movement/transport would essentially play an important role in lysosomal acidification and cell proliferation *via* autophagy. However, it has not been confirmed that the functional presence of Cl^−^ transporter, ClC-7, is essentially required for lysosomal acidification and autophagy function. Namely, there are no direct evidence indicating that the presence and movement/transport of Cl^−^ are essentially required for lysosomal acidification and autophagy function. In other words, it is still unclear if the presence of Cl^−^ itself as a target ion transported by ClC-7 plays an essential role in lysosomal acidification and autophagy function.

Our previous reports indicated that Cl^−^ plays various important roles in cellular functions; namely, lowering cytosolic Cl^−^ inhibits proliferation of cancer cells [18–26] and elongation of neurite in neuronal cells [27–31], but activates expression of epithelial Na^+^ channel [32–34] and Na^+^-permeant channel [35]. Thus, we tried to clarify the role of Cl^−^ in acidification of lysosome and function of autophagy in the present study by using a model cancer cell line (MKN28) by replacing Cl^−^ with NO_3_^−^, which generally has permeability identical to Cl^−^ in Cl^−^ channels.

## Materials and methods

### Materials

Roswell Park Memorial Institute (RPMI) 1640 medium, bafilomycin A1 (an inhibitor of V-type H^+^-ATPase), ethyl isopropyl amiloride [EIPA; an inhibitor of Na^+^/H^+^ exchanger (NHE)], acridine orange (AO) and valinomycin were purchased from Sigma-Aldrich (St. Louis, MO, USA). *N*-(Ethoxycarbonyl methyl)-6-methoxy quinolinium (MQAE) and carboxy-seminaphthorhodafluor-1 (carboxy-SNARF-1) were obtained from Invitrogen (Carlsbad, CA, USA). Nigericin, tributyltin chloride and monensin were purchased from Wako Pure Chemical Industries (Osaka, Japan). The moderately differentiated human gastric adenocarcinoma cell line, MKN28, was obtained from ATCC (Manassas, VA, USA).

### Culture conditions

MKN28 cells were seeded into 25 cm^2^ flasks at a density of 2.5 × 10^5^ cells/flask and incubated for 24 hrs in RPMI1640 medium (Sigma-Aldrich) supplemented with 5% foetal bovine serum (FBS) in a humidified incubator at 37°C with 5% CO_2_ in air. We defined this time-point as time zero (0 hr) at 24 hrs culture after seeding. Cells were then cultured for 48 hrs under six different conditions: (1) RPMI medium as control, (2) RPMI medium with a low Cl^−^ concentration (low Cl^−^ medium; low Cl^−^ condition), (3) RPMI medium with a low pH (low pH medium; low Cl^−^ condition), (4) RPMI medium containing bafilomycin A1, (5) RPMI medium containing EIPA and (6) RPMI containing dimethyl sulfoxide (DMSO) as solvent control for bafilomycin A1 and EIPA. For RPMI medium with a low Cl^−^ concentration, we used a Cl^−^-replaced RPMI1640 medium (Cell Science and Technology Institute, Sendai, Japan) prepared by substituting Cl^−^ with NO_3_^−^, as previously reported [20]. For RPMI with a low pH, we adjusted pH of medium by adding HNO_3_ to pH 6.9. The concentrations of bafilomycin A1, EIPA and DMSO (solvent control for bafilomycin A1 and EIPA) were respectively 1 nM, 25 μM and 0.1% as previously reported [18].

### Measurement of cytosolic pH (pH_c_)

Cells were seeded into 35 mm glass bottom dishes at a density of 1.0 × 10^5^ cells/dish and incubated for 24 hrs in RPMI1640 medium supplemented with 5% FBS in a humidified incubator at 37°C with 5% CO_2_ in air followed by culture under six different conditions for 48 hrs in a humidified incubator at 37°C with 5% CO_2_ in air. We measured pH_c_ of MKN28 cells under each condition by using carboxy-SNARF-1, a pH-sensitive fluorescent dye, with an inverted confocal laser microscope, LSM510 META (Carl Zeiss, Jena, Germany) at 37°C in air with 5% CO_2_ as previously reported [18]. The emission ratio was calibrated by using solutions (110 mM KCl, 25 mM KHCO_3_, 11 mM glucose, 1 mM MgCl_2_, 1 mM CaCl_2_, 10 mM HEPES) at 37°C in air with 5% CO_2_ with varying pH adjusted by CsOH that contained 10 μM nigericin (K^+^/H^+^ ionophore). The fluorescence emission ratio (645 nm/592 nm) was calculated and used to estimate the pH_c_ under each condition from the calibration curve. We evaluated the mean value of pH_c_ from more than 30 cells in one culture dish. We then treated this mean value of pH_c_ as ‘n = 1’. Next, with the same procedure, we obtained the mean value of pH_c_ from different culture dishes. By using the mean values of pH_c_ obtained from different cell culture dishes, we calculated the mean value of pH_c_ shown in the present study. Namely, ‘n’ shown in the present study means the number of cell culture dishes used for measurement of pH_c_.

### Measurement of lysosomal pH (pH_lys_)

We cultured cells by using the condition same as measurement of cytosolic pH. We measured pH_lys_ of MKN28 cells cultured under each condition at 37°C with 5% CO_2_ incubation, by using LysoSensorTM Yellow/Blue DND-160 (Invitrogen) with an inverted confocal laser microscope, LSM510META (Carl Zeiss). LysoSensor-loaded cells were washed with each conditioning medium, and incubated for 20 min. at 37°C. The intensity of LysoSensor in the chamber on the stage of the microscope (ZILCS) was then measured by LSM510 META system. The sample was excited at 780 nm with a 2-photon excitation laser system (MaiTai®, Spectra-Physics, Tokyo, Japan). The laser beam was directed to the dish containing the cells *via* a C-Apochromat 40× water-immersion objective lens (Carl Zeiss). The emitted fluorescence was simultaneously collected by a gating, and the separated fluorescence was detected by 24 PMTs. We collected two PMTs at 540 and 440 nm. The intensity of fluorescence was digitized with a META system. Several regions of interest (ROI) were then randomly selected. The emission ratio was calibrated by using solutions (115 mM KCl, 5 mM NaCl, 1 mM MgCl_2_, 25 mM MES) with varying pH (pH from 3.5 to 6) that contained 10 μM nigericin (K^+^/H^+^ ionophore) and 10 μM monensin (Wako Pure Chemical Industries). The fluorescence emission ratio (440 nm/540 nm) was calculated and used to estimate pH_lys_ from the calibration curve. To evaluate the mean value of pH_lys_, we applied the experimental procedure same as pH_c_ measurement. The meaning of ‘n’ is same as that of pH_c_.

### Measurement of cytosolic chloride ion concentration ([Cl^−^]_c_)

We cultured cells by using the condition same as measurement of cytosolic pH. We measured [Cl^−^]_c_ of MKN28 cells cultured under each different condition by using MQAE at 37°C in air with 5% CO_2_ with an inverted confocal laser microscope, LSM510 META (Carl Zeiss) as previously reported [18]. The sample was excited at 780 nm for MQAE by using a 2-photon excitation laser system (MaiTai®, Spectra-Physics). The emitted fluorescence was simultaneously collected by a gating, and the separated fluorescence was detected by 24 PMTs. We collected one PMT at 460 nm for MQAE. MQAE is insensitive to pH changes within physiological ranges. The intensity of fluorescence was digitized with a META system. Several ROI were then randomly selected excluding vesicles and nuclear regions. Calibration of the fluorescence based on [Cl^−^]_c_ was accomplished by 5 μM nigericin, 5 μM valinomycin (K^+^ ionophore) and 10 μM tributyltin chloride (Cl^−^/OH^−^ ionophore) in 140 mM-K^+^ calibration buffers (pH 7.4) with various Cl^−^ concentrations. The ionophores were used to adjust the [Cl^−^]_c_ to a level identical to the extracellular Cl^−^ concentration at constant cytosolic K^+^ and H^+^ concentrations. To evaluate the mean value of [Cl^−^]_c_, we applied the experimental procedure same as pH_c_ measurement. The meaning of ‘n’ is same as that of pH_c_.

### Measurement of lysosomal Cl^−^ concentration ([Cl^−^]_lys_)

We cultured cells by using the condition same as measurement of cytosolic pH. Cells cultured in a glass bottom dish with the culture medium were stained with MQAE and a lysosome-detectable dye, AO, accumulating into lysosomes in each culture medium containing 5 mM MQAE and 3 μM AO for 30 min. in a humidified incubator at 37°C with 5% CO_2_ in air. We measured [Cl^−^]_lys_ of MKN28 cells under each condition by detecting MQAE fluorescence at 37°C in air with 5% CO_2_ by using an inverted confocal laser microscope, LSM510 META (Carl Zeiss). The sample was excited at 780 nm for MQAE by using a 2-photon excitation laser system (MaiTai®, Spectra-Physics). The emitted fluorescence was then simultaneously collected by a gating, and the separated fluorescence was detected by 24 PMTs. We collected one PMT at 460 nm for MQAE. To detect lysosomal areas, the sample was excited at 488 nm by using an Ar laser for measurement of AO fluorescence and the emitted fluorescence was simultaneously collected by a gating, and the separated fluorescence was detected by 24 PMTs. We collected one PMT at 640 nm for AO fluorescence. The intensity of MQAE in lysosome detected by AO fluorescence in the chamber on the stage of the microscope (ZILCS) was measured by the LSM510 META system. The intensity of fluorescence was digitized with a META system. Several ROI in lysosomal areas were then randomly selected. Calibration of the fluorescence based on [Cl^−^]_lys_ was accomplished by 5 μM nigericin, 5 μM valinomycin and 10 μM tributyltin chloride in 140 mM-K^+^ calibration buffers (pH 7.4) with various Cl^−^ concentrations to determine the [Cl^−^]_lys_ under each condition. To evaluate the mean value of [Cl^−^]_lys_, we applied the experimental procedure same as pH_c_ measurement. The meaning of ‘n’ is same as that of pH_c_.

### Assay of cell proliferation

After culture under each experimental condition for 48 hrs, cells were detached from the flasks with trypsin-ethylenediaminetetraacetic acid (EDTA) and directly counted with a haematocytometer.

### Analysis of cell cycle

After culturing the cells in each conditioning medium for 48 hrs, we detached the cells from the flasks with trypsin-EDTA treatment, and centrifuged the detached cells. Nuclear Isolation Medium of 0.5 ml (NIM-DAPI 10; Beckman Coulter, Fullerton, CA, USA) was added to cells in the pellets. We determined cell cycle phases from 10,000 cells by using FlowJo software (Tree Star Inc., Ashland, OR, USA) by the Cell Lab Quanta (Beckman Coulter) with an excitation at 365 nm and emission at 450 nm for DAPI.

### Western blotting

Immunoblotting method to detect following proteins was performed under each condition as previously reported [18,36]. The blots were incubated with a primary anti-p62 antibody (Medical and Biological Laboratories Co., Nagoya, Japan), anti-LC3 (Sigma-Aldrich), anti-phospho-Akt (Ser473), or anti-total Akt, anti-cleaved caspase 3, anti-cleaved caspase 9 or anti-GAPDH antibody (Cell Signaling Technology, Beverly, MA, USA). All proteins were detected by using the ECL plus (GE Healthcare, Buckinghamshire, UK). We measured the band densities with Image Lab (Bio-Rad, Hercules, CA, USA) after scanning with Chemidoc XRS Plus system (Bio-Rad). We detected the expression of these proteins in the cells we cultured at same time when we cultured the cells for measurements of pH_c_, pH_lys_, [Cl^−^]_c_ and [Cl^−^]_lys_. These experimental procedures indicate that the cells used for detection of the protein expression stayed among the cell population same as that used for measurements of pHc and pH_lys_.

### Statistical analysis

Data are expressed as means ± SEM. Statistical significance was determined by using a multiple comparison test (Tukey–Kramer). The differences were considered significant when the *P* < 0.05.

## Results

### Cytosolic proton concentration (pH_c_)

**Figure 1:**
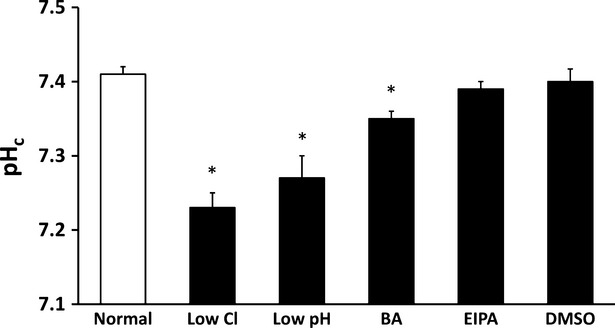
Measurement of pH_c_ in MKN28 cells exposed to low Cl^−^ condition, low pH condition, bafilomycin A1 (BA), ethyl isopropyl amiloride (EIPA) or DMSO alone as solvent control for bafilomycin A1 or EIPA. Low Cl^−^ condition, low pH condition and bafilomycin A1 caused significant decreases of pH_c_ in MKN28 cells; *n* = 4, *P < 0.05.

Cell culture in low Cl^−^ medium significantly decreased pH_c_ compared with that in normal medium (Fig.1). As low Cl^−^ condition led pH_c_ to a lowered level, we tried to mimic the effect of low Cl^−^ condition on pH_c_ by culturing cells in low pH medium expected to decrease pH_c_. As expected, pH_c_ of cells cultured in low pH medium was almost identical to that in low Cl^−^ medium, which was much lower than that in normal medium. Bafliomycin A1 slightly decreased pH_c_, but its effect on pH_c_ was much smaller than that in low Cl^−^ or pH medium (Fig.1). Ethyl isopropyl amiloride had no significant effect on pH_c_ (Fig.1). Application of DMSO alone (solvent control for bafilomycin A1 and EIPA) had no significant effects on pH_c_ compared with normal condition (Fig.1).

### Lysosomal pH (pH_lys_)

**Figure 2:**
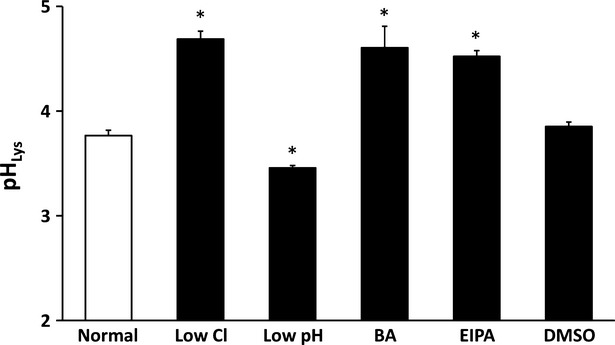
Measurement of pH_lys_ in MKN28 cells exposed to low Cl^−^ condition, low pH condition, bafilomycin A1 (BA), ethyl isopropyl amiloride (EIPA) or DMSO alone. Low Cl^−^ condition, bafilomycin A1 and EIPA caused significant increases of pH_lys_ in MKN28 cells. Low pH condition significantly caused a decrease of pH_lys_ in MKN28 cells; *n* = 4, *P < 0.05.

As shown in Figure2, cells cultured in normal medium showed pH_lys_ a little bit lower than 4.0, and application of DMSO alone had no significant effect on pH_lys_. pH_lys_ in low Cl^−^ medium was much higher than that in normal medium (Fig.2). However, although low pH condition decreased pH_c_ similar to low Cl^−^ condition, pH_lys_ in low pH medium was lower than that in normal medium unlike low Cl^−^ condition (Fig.2). Application of bafilomycin A1 or EIPA increased pH_lys_ to a level identical to that in low Cl^−^ medium (Fig.2).

### Cytosolic chloride ion concentration ([Cl^−^]_c_)

**Figure 3:**
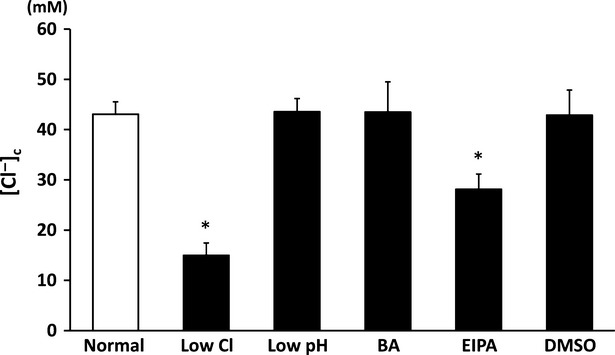
Measurement of [Cl^−^]_c_ in MKN 28 cells exposed to low Cl^−^ condition, low pH condition, bafilomycin A1 (BA), ethyl isopropyl amiloride (EIPA) or DMSO alone. Low Cl^−^ condition and EIPA significantly decreased [Cl^−^]_c_ in MKN28 cells. Low pH, bafilomycin A1 (BA) or DMSO caused no significant change in [Cl^−^]_c_ in MKN28 cells; *n* = 4, *P < 0.05.

As shown in Figure3, [Cl^−^]_c_ of cells cultured in low Cl^−^ medium was much lower than that in normal medium; application of DMSO alone had no significant effects on [Cl^−^]_c_ compared with normal condition (Fig.3). Furthermore, [Cl^−^]_c_ of cells treated with EIPA was significantly lower than that in normal or DMSO-containing medium, however the EIPA-induced decrease in [Cl^−^]_c_ was much smaller than that induced by culture in a low Cl^−^ medium. On the other hand, low pH or bafilomycin A1 had no significant effects on [Cl^−^]_c_ compared with that in normal or DMSO-containing medium (Fig.3).

### Lysosomal chloride ion concentration ([Cl^−^]_Lys_)

**Figure 4:**
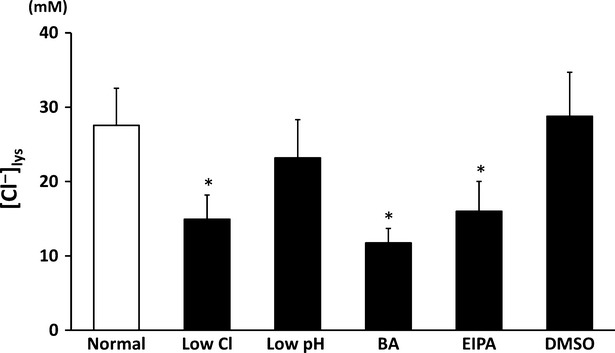
Measurement of Cl^−^ concentration in lysosome ([Cl^−^]_lys_) in MKN28 cells exposed to low Cl^−^ condition, low pH condition, bafilomycin A1 (BA), ethyl isopropyl amiloride (EIPA) or DMSO alone. Low Cl^−^ condition, bafilomycin A1 (BA) and EIPA, but not low pH condition caused significant decreases of [Cl^−^]_lys_ in MKN28 cells; *n* = 3, *P < 0.05.

[Cl^−^]_Lys_ significantly decreased in low Cl^−^ medium compared with that in normal or DMSO-containing medium (Fig.4); DMSO alone had no significant effect on [Cl^−^]_Lys_ (Fig.4). Bafilomycin A1 or EIPA decreased [Cl^−^]_Lys_ to a level identical to that in low Cl^−^ medium (Fig.4). Low pH medium had no significant effect on [Cl^−^]_Lys_ (Fig.4).

### Cell proliferation

**Figure 5:**
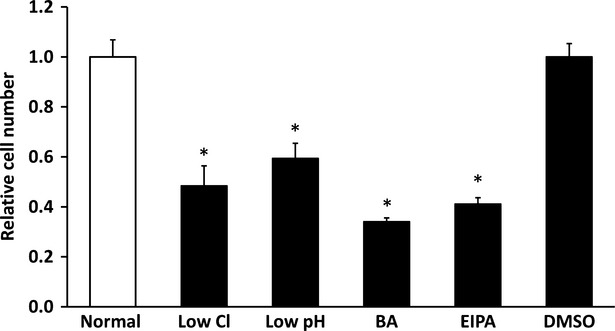
Cell proliferation under low Cl^−^ condition, low pH condition, bafilomycin A1 (BA), ethyl isopropyl amiloride (EIPA) or DMSO alone. The number of cells cultured under control condition is expressed as 1. Proliferation of cells was significantly suppressed by low Cl^−^ condition, low pH condition, bafilomycin A1 (BA) or EIPA compared with normal condition or DMSO alone; *n* = 6, *P < 0.05.

Cell proliferation of MKN28 cells cultured in low Cl^−^, low pH, bafilomycin A1 or EIPA condition was significantly diminished (Fig.5).

### Cell cycle analysis

**Figure 6:**
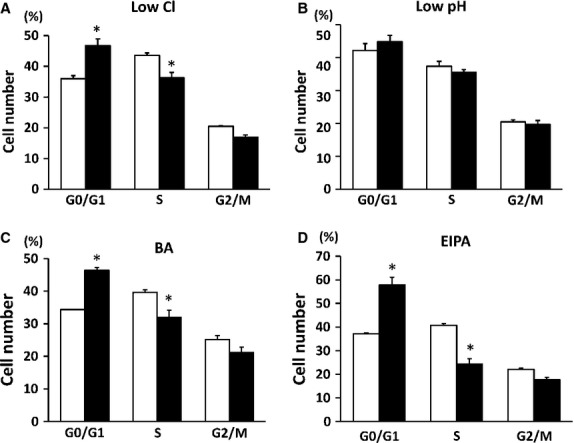
The percentage of MKN28 cells staying in each phase of the cell cycle in low Cl^−^ condition (A), low pH condition (B), bafilomycin A1 (BA; C) or ethyl isopropyl amiloride (EIPA; D). Open columns indicate the percentage of MKN28 cells cultured under normal condition for low Cl^−^ condition (A) and low pH condition (B) or DMSO alone for BA (C) and EIPA (D). The closed column indicates the percentage of MKN28 cells cultured under low Cl^−^ condition (A), low pH condition (B), BA (C) and EIPA (D). The population of cells in the G_0_/G_1_ phase was increased and that in the S phase was significantly decreased by exposure to low Cl^−^ condition (A), BA (C), or EIPA (D) compared with normal condition for low Cl^−^ condition or DMSO alone for BA or EIPA. Low pH (B) condition caused no significant change in cell cycle compared with normal condition; *n* = 6, *P < 0.05.

As mentioned above, low Cl^−^, low pH, bafilomycin A1 or EIPA condition diminished cell proliferation. Therefore, we analysed cell cycle under these conditions. Cell cycle analysis indicates that application of low Cl^−^ medium, bafilomycin A1 or EIPA significantly increased the G_0_/G_1_ fraction associated with reduction of the S-phase fraction (Fig.6). However, although low pH condition diminished cell proliferation, low pH condition has no significant effect on any cell cycle phases (Fig.6). These observations suggest that application of low Cl^−^, bafilomycin A1 or EIPA induces G_0_/G_1_ arrest, but that low pH condition induces no specific arrest of cell cycle (Fig.6).

### Autophagy-related proteins

**Figure 7:**
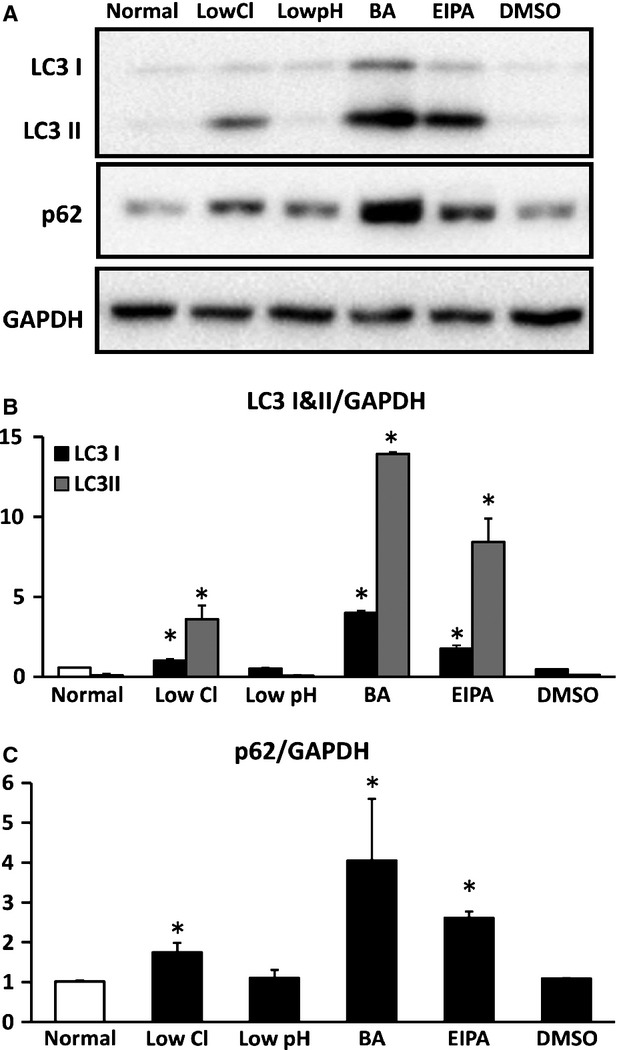
Expression of LC3 I, LC3 II and p62. (A) Expression of LC3 I and II, p62 and GAPDH by Western blotting. (B) Quantitative results of expression of LC3 I and LC3 II, and p62 normalized by GAPDH with Western blotting. Low Cl^−^ condition, bafilomycin A1 (BA) or ethyl isopropyl amiloride (EIPA), but not low pH condition, caused significant increases of LC3 I and II compared with those under the normal condition. (C) Quantitative results of expression of p62 normalized by GAPDH with Western blotting. Low Cl^−^ condition, BA or EIPA, but not low pH condition caused a significant increase in p62 compared with normal condition for low Cl^−^ condition and low pH condition or DMSO for BA and EIPA in MKN28 cells cultured for 48 hrs; *n* = 4, *P < 0.05.

Markers of autophagy degrading function, LC3 I, LC3 II and p62 [37,38], were detected under each different condition. Application of low Cl^−^, bafilomycin A1 or EIPA significantly elevated amounts of those proteins compared with those under normal or DMSO-containing medium, but low pH condition did not significantly affect amounts of those proteins (Fig.7).

### Apoptosis-related proteins

**Figure 8:**
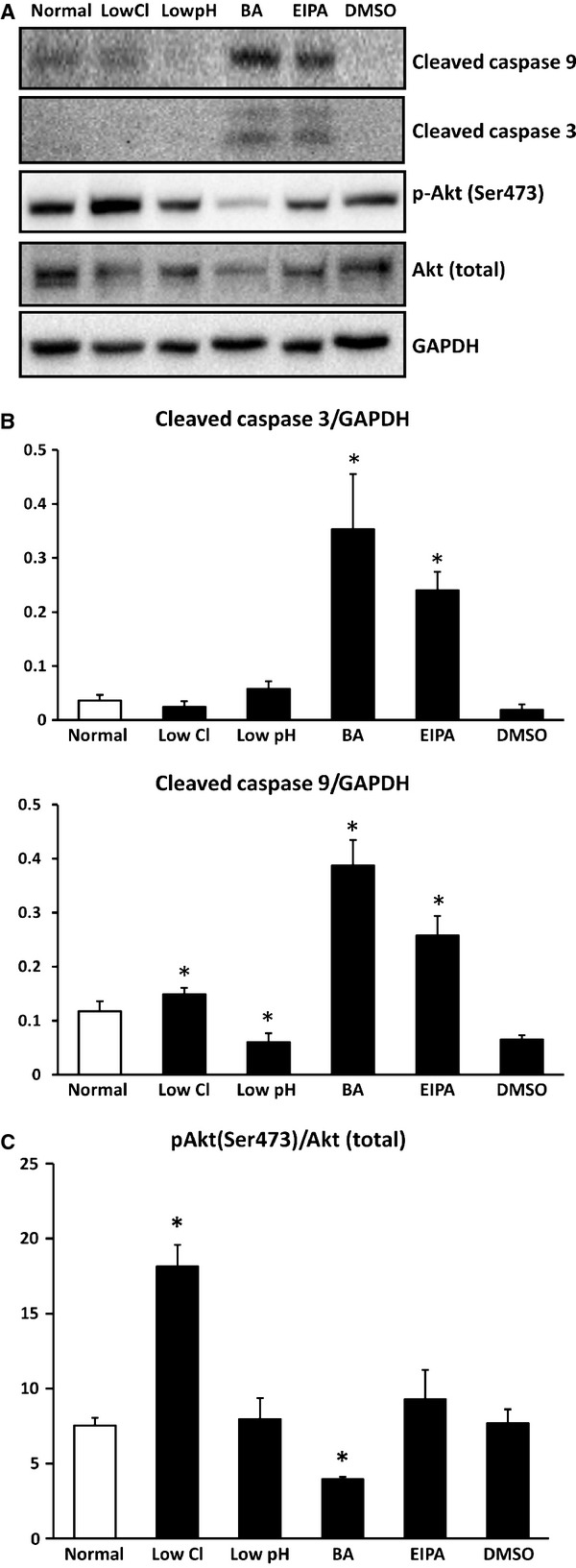
Expression of cleaved caspase3, cleaved caspase9, Akt (total), phospho-Akt (Ser473) and GAPDH. (A) Expression of cleaved caspase3, cleaved caspase9, Akt (total), phospho-Akt (Ser473) and GAPDH by Western blotting. (B) Low Cl^−^ condition caused a significant increase in cleaved-caspase9 compared with normal condition in MKN28 cells without any effect on cleaved-caspase3. On the one hand, low pH condition caused a significantly decrease in cleaved-caspase9 compared with normal condition in MKN28 cells without any effect on cleaved-caspase3. Bafilomycin A1 (BA) or ethyl isopropyl amiloride caused significant increases in both cleaved-caspase3 and cleaved-caspase9 compared with DMSO alone in MKN28 cells. (C) Low Cl^−^ condition caused a significant increase in the ratio of phospho-Akt/Akt (total) compared with normal condition in MKN28 cells. On the other hand, BA caused a significant decrease in the ratio of phospho-Akt/Akt (total) compared with DMSO alone in MKN28 cells; *n* = 4, *P < 0.05.

Expression of cleaved caspase 3 and 9, apoptosis-related proteins, drastically increased in cells treated with bafilomycin A1 or EIPA compared with those in normal or DMSO-containing medium (Fig.8A and B). In low Cl^−^ or low pH medium, we detected no significant change in expression of cleaved caspase 3 compared that in normal or DMSO-containing medium (Fig.8A and B). We also observed that expression of caspase 9 was slightly increased by low Cl^−^ condition but decreased by low pH, however the effect of low Cl^−^ or pH on expression of cleaved caspase 3 and 9 was much less than that of bafilomycin A1 or EIPA (Fig.8A and B). These observations suggest that apoptosis would be induced by application of bafilomycin A1 or EIPA, but not by low Cl^−^ or pH condition. Taken together, these observations shown in Figures7 and 8 suggest that impairment of autophagy would not be functionally correlated with apoptosis.

Phosphorylated Akt, active form of Akt, is well known to prevent apoptosis *via* several pathways [39]. Phosphorylated Akt (Ser473) in low Cl^−^ medium significantly increased compared with that in normal medium, but not under other conditions (Fig.8A and C). These observations suggest that apoptosis would be prevented by lowering [Cl^−^]_c_ *via* an increase in phosphorylated Akt, and that low Cl^−^ condition diminishes cell proliferation without induction of apoptosis.

## Discussion

Our present study focused on clarifying roles of cytosolic Cl^−^ in lysosomal acidification required for autophagy. Our observations shown in the present study indicate that cell culture in low Cl^−^ medium decreased [Cl^−^]_c_ with lowered pH_c_, and also increased pH_lys_ with decreased [Cl^−^]_lys_, leading cells to dysfunction of autophagy *via* impairment of lysosomal digestion and G_0_/G_1_ arrest-caused inhibition of cell growth without apoptosis. It is suggested that the presence of Cl^−^ itself but not ClC-7 itself might be essentially required for lysosomal acidification and autophagy function.

**Figure 9:**
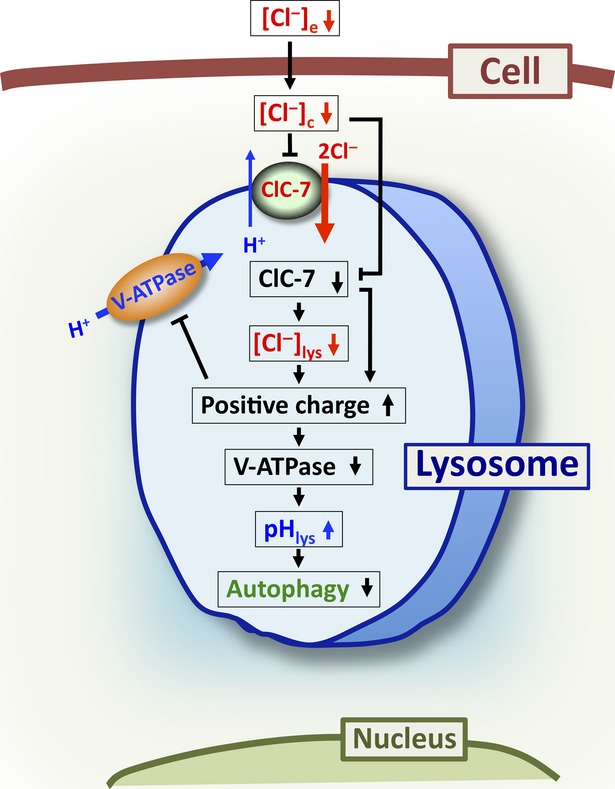
Action of low Cl^−^ on lysosomal function. (1) lowered Cl^−^ concentration of extracellular space ([Cl^−^]_e_) decreases [Cl^−^]_c_ ([Cl^−^]_e_↓ → [Cl^−^]_c_↓); (2) lowered [Cl^−^]_c_ diminishes activity of ClC-7 ([Cl^−^]_c_↓ → ClC-7↓); (3) lowered activity of ClC-7 decreases [Cl^−^]_lys_ (ClC-7↓ → [Cl^−^]_lys_↓); (4) both lowered ClC-7 activity and [Cl^−^]_lys_ elevate the amount of positive charges remaining in intra-lysosomal space (ClC-7↓/[Cl^−^]_lys_↓ → positive charges in lysosome↑); (5) elevation of amount of positive charges remaining in intra-lysosomal space diminishes transporting activity V-ATPase (V-type H^+^-ATPase) *via* an increase in electrical barrier for V-ATPase transporting a positive charge against the electrical barrier across the lysosomal membrane (positive charges in lysosome↑ → V-ATPase↓); (6) lowered activity of V-ATPase induces an increase in pH_lys_ (a decrease in H^+^ concentration intra-lysosomal space; V-ATPase↓ → pH_lys_↑) and (7) lowered pH_lys_ disturbs autophagy function (V-ATPase↓ → autophagy↓).

The mechanism of low pH_c_ induced by culture in low Cl^−^ medium would be because of lowered activity of NHE and/or Na^+^-driven Cl^−^/bicarbonate (HCO_3_^−^) exchanger (NDCBE) participating in HCO_3_^−^ uptake into cytosolic space caused by lowered [Cl^−^]_c_, as NHE activity is diminished by lowering [Cl^−^]_c_ [40], and NDCBE activity also depends on [Cl^−^]_c_ [41]. The mechanism increasing pH_lys_ observed in low Cl^−^ medium would be because of an insufficient amount of counter anion, Cl^−^, in the cytosolic space (lowered [Cl^−^]_c_) co-transported into the intra-luminal space at proton moving into intra-lysosomal space disturbing proton movement into intra-lysosomal space. Thus, an insufficient amount of cytosolic Cl^−^ would cause elevation of pH_lys_ associated with low [Cl^−^]_lys_, and NO_3_^−^ would not play an identical role to Cl^−^ in lysosomal acidity or autophagy function (see the conclusion and Fig.9 in detail).

To maintain acidic conditions of intra-lysosomal space is very important for function of lysosomal digestion mediated by more than 70 kinds of enzymes such as proteinase, lipase, nuclease *etc*. In autophagy, proteinases including cathepsin B, D and L [42–44] are potent enzymes to degrade proteins in lysosomes. The pH maintaining optimal activity of these cathepsins ranges from 3.0 to 4.0 [45–48]. This means that increased pH_lys_ higher than 4.0 might diminish activity of lysosomal digestion of proteins, leading to disturbance of autophagy. Application of EIPA had an action on pH_lys_ and [Cl^−^]_lys_ similar to bafilomycin A1; *i.e*. each compound significantly increased pH_lys_ to a level higher than 4.0 associated with a decrease in [Cl^−^]_lys_, causing dysfunction of autophagy. However, the mechanism of pH_lys_ increased by EIPA is different from that by bafilomycin A1, which primarily inhibits proton uptake into the intra-lysosomal space. As we have previously reported, EIPA primarily causes a decrease in [Cl^−^]_c_ by inhibiting NHE [18] unlike primary inhibition of H^+^ movement by bafilomycin A1. This decreased [Cl^−^]_c_ would diminish [Cl^−^]_lys_ associated with increased pH_lys_ *via* a mechanism similar to that under the low Cl^−^ condition, although the effect of EIPA was smaller than that of low Cl^−^ condition. Based on these observations shown in the present study, it is clear that lowered [Cl^−^]_c_ has potential to increase pH_lys_, disturbing autophagy because of elevated pH_lys_-induced inhibition of enzymatic digestion in lysosomes.

In the present study, we indicate that the proliferation of MKN28 cells was diminished under each tested condition except DMSO alone. In cell cycle analysis, low Cl^−^ condition, bafilomycin A1 or EIPA induced G_0_/G_1_ arrest, whereas low pH condition inhibited proliferation without any specific cell cycle arrest. We have already reported that lowered [Cl^−^]_c_ causes G_0_/G_1_ arrest in MKN28 cells *via* activation of MAP kinase [18,36]. It has been demonstrated that decreased pH_c_ elevates the expression of checkpoint proteins for cell cycle (*e.g*. cyclinB1 and cdc2) or signal transduction-related proteins (*e.g*. Wee1 kinase) [49]. However, the present study shows that low pH condition did not induce any specific cell cycle arrest, but would cause slow transition rates at all phases of cell cycle or cell stemness similar to an observation that low pH condition induces dormancy in cancer cells [50].

Low Cl^−^ condition caused increases in LC3 II and p62 (Fig.7). Inhibition of autophagy leads to an increase in p62 [51], while the amount of LC3 II is correlated with the number of autophagosomes [52]. The observations on low Cl^−^-caused increases in LC3 II and p62 look contradictory. However, it is also reported that LC3 II itself is degraded by autophagy 53; *i.e*. impairment of autophagy elevates the amount of LC3 II by inhibiting the degradation of LC3 II [53]. The increase in both LC3 II and p62 observed under low Cl^−^ condition would be because of impairment of autophagy, suggesting that low Cl^−^ condition would impair autophagy.

It is clarified that knock down of Atg5 or Atg7 causes impairment of autophagy system associated with apoptosis of cancer cells and inhibition of cell growth [11–13]. However, the relationship between induction of apoptosis and impairment of autophagy still is unclear. Amaravadi *et al*. [11] and Guo *et al*. [12] report that impairment of autophagy induces caspase-dependent apoptosis. On the other hand, Racoma *et al*. [54] report that a lysosome acidification inhibitor, weak base, induces caspase-independent apoptosis associated with dysfunction of autophagy *via* release of cathepsin B from lysosome. The present study indicates that each application of low Cl^−^ condition, bafilomycin A1 or EIPA caused dysfunction of autophagy. On the one hand, increased expression of caspase3 and caspase9 related to apoptosis was observed in cases of bafilomycin A1 and EIPA, but not under low Cl^−^ condition. Furthermore, bafilomycin A1 inhibited phosphorylation of Akt, which could accelerate apoptosis. On the other hand, low Cl^−^ condition could prevent apoptosis by enhancing phosphorylation of Akt. These observations suggest that: (*i*) both low Cl^−^ condition and application of bafilomycin A1 inhibited cell growth associated with dysfunction of autophagy; (*ii*) the inhibition of cell growth caused by low Cl^−^ condition would not induce apoptosis *via* enhancement of Akt phosphorylation, but bafilomycin A1-caused inhibition of cell growth would induce apoptosis *via* diminution of Akt phosphorylation. Akt is a key regulator of a variety of proteins involved in cell proliferation, survival and apoptosis. The PI3K/Akt pathway is important in cancer cells to survive, proliferate and prevent apoptosis. Phosphorylated Akt, the active form of Akt, is well known to prevent apoptosis *via* several pathways [39,55]. Phosphorylated Akt in low Cl^−^ medium significantly increased compared with that in normal medium, but not under other conditions. These observations suggest that apoptosis would be prevented by lowering [Cl^−^]_c_ *via* an increase in phosphorylated Akt, and that low Cl^−^ condition diminishes cell proliferation without induction of apoptosis. However, it is still unclear whether the low Cl^−^-induced impairment of autophagy performs cross-talk to phosphorylation of Akt or occurs in parallel with phosphorylation of Akt. Further studies are required to clarify the relationship between autophagy dysfunction and phosphorylation of Akt, although low Cl^−^ condition could prevent apoptosis by enhancing phosphorylation of Akt.

## Conclusions

Observations shown in the present study suggest the following points (Fig.9): (*i*) lowered Cl^−^ concentration of extracellular space ([Cl^−^]_e_) decreases [Cl^−^]_c_; (*ii*) lowered [Cl^−^]_c_ diminishes activity of ClC-7; (*iii*) lowered activity of ClC-7 with exchange of 2Cl^−^/1H^+^ diminishes [Cl^−^]_lys_; (*iv*) both lowered activity of ClC-7 and [Cl^−^]_lys_ elevate the amount of positive charges remaining in intra-lysosomal space; (*v*) elevation of amount of positive charges remaining in intra-lysosomal space diminishes activity V-type H^+^-ATPase *via* an increase in electrical barrier for V-type H^+^-ATPase transporting a positive charger against the electrical barrier across the lysosomal membrane; (*vi*) lowered activity of V-type H^+^-ATPase induces an increase in pH_lys_ (a decrease in H^+^ concentration intra-lysosomal space) and (*vii*) lowered pH_lys_ disturbs autophagy function.

## References

[1] He C, Klionsky DJ (2009). Regulation mechanisms and signaling pathways of autophagy. Annu Rev Genet.

[2] Kroemer G, Marino G, Levine B (2010). Autophagy and the integrated stress response. Mol Cell.

[3] Mizushima N, Komatsu M (2011). Autophagy: renovation of cells and tissues. Cell.

[4] Grisolia S, Knecht E, Hernandez-Yago J (1979). Turnover and degradation of mitochondria and their proteins. Ciba Found Symp.

[5] Kawai A, Uchiyama H, Takano S (2007). Autophagosome-lysosome fusion depends on the pH in acidic compartments in CHO cells. Autophagy.

[6] Youle RJ, Narendra DP (2011). Mechanisms of mitophagy. Nat Rev Mol Cell Biol.

[7] Di X, Zhang G, Zhang Y (2013). Accumulation of autophagosomes in breast cancer cells induces TRAIL resistance through downregulation of surface expression of death receptors 4 and 5. Oncotarget.

[8] Yang L, Li P, Fu S (2010). Defective hepatic autophagy in obesity promotes ER stress and causes insulin resistance. Cell Metab.

[9] Fritz T, Niederreiter L, Adolph T (2011). Crohn's disease: NOD2, autophagy and ER stress converge. Gut.

[10] White E (2012). Deconvoluting the context-dependent role for autophagy in cancer. Nat Rev Cancer.

[11] Amaravadi RK, Yu D, Lum JJ (2007). Autophagy inhibition enhances therapy-induced apoptosis in a Myc-induced model of lymphoma. J Clin Invest.

[12] Guo JY, Chen HY, Mathew R (2011). Activated Ras requires autophagy to maintain oxidative metabolism and tumorigenesis. Genes Dev.

[13] Yang S, Wang X, Contino G (2011). Pancreatic cancers require autophagy for tumor growth. Genes Dev.

[14] Graves AR, Curran PK, Smith CL (2008). The Cl^−^/H^+^ antiporter ClC-7 is the primary chloride permeation pathway in lysosomes. Nature.

[15] Leisle L, Ludwig CF, Wagner FA (2011). ClC-7 is a slowly voltage-gated 2Cl^−^/1H^+^-exchanger and requires Ostm1 for transport activity. EMBO J.

[16] Weinert S, Jabs S, Supanchart C (2010). Lysosomal pathology and osteopetrosis upon loss of H^+^-driven lysosomal Cl^−^ accumulation. Science.

[17] Lange PF, Wartosch L, Jentsch TJ (2006). ClC-7 requires Ostm1 as a beta-subunit to support bone resorption and lysosomal function. Nature.

[18] Hosogi S, Miyazaki H, Nakajima K (2012). An inhibitor of Na^+^/H^+^ exchanger (NHE), ethyl-isopropyl amiloride (EIPA), diminishes proliferation of MKN28 human gastric cancer cells by decreasing the cytosolic Cl^−^ concentration *via* DIDS-sensitive pathways. Cell Physiol Biochem.

[19] Hosogi S, Ohta M, Nakajima K (2012). The mechanisms of Na^+^/H^+^ exchanger inhibitor on proliferation of gastric cancer cells with several pathways. J Physiol Sci.

[20] Miyazaki H, Shiozaki A, Niisato N (2008). Chloride ions control the G_1_/S cell-cycle checkpoint by regulating the expression of p21 through a p53-independent pathway in human gastric cancer cells. Biochem Biophys Res Commun.

[21] Miyazaki H, Niisato N, Marunaka Y (2013). Oscillatory changes in the activity of Na^+^-K^+^-2Cl^−^ cotransporter control cell cycle progression *via* changes in the intracellular concentration of Cl- in MKN28 human gastric cancer cells. J Physiol Sci.

[22] Hiraoka K, Miyazaki H, Niisato N (2010). Chloride ion modulates cell proliferation of human androgen-independent prostatic cancer cell. Cell Physiol Biochem.

[23] Hosogi S, Miyazaki H, Kusuzaki K (2013). Cl^−^ channels/transporters as new targets for cancer therapies based on disruption of autophagy ability *via* modification of lysosome acidification. J Physiol Sci.

[24] Kitagawa M, Niisato N, Hosogi S (2013). A role of K^+^-Cl^−^ cotransporter in the cell cycle regulation of breast cancer MDA-MB-231 cells. J Physiol Sci.

[25] Shiozaki A, Miyazaki H, Niisato N (2006). Furosemide, a blocker of Na^+^/K^+^/2Cl^−^ cotransporter, diminishes proliferation of poorly differentiated human gastric cancer cells by affecting G0/G1 state. J Physiol Sci.

[26] Kitagawa M, Niisato N, Shiozaki A (2013). A regulatory role of K^+^-Cl^−^ cotransporter in the cell cycle progression of breast cancer MDA-MB-231 cells. Arch Biochem Biophys.

[27] Nakajima K, Nagao H, Niisato N (2011). The role of potassium chloride cotransporter 1 in neurite outgrowth of PC12 cell. J Physiol Sci.

[28] Nakajima K, Niisato N, Marunaka Y (2011). Quercetin stimulates NGF-induced neurite outgrowth in PC12 cells *via* activation of Na^+^/K^+^/2Cl^−^ cotransporter. Cell Physiol Biochem.

[29] Nakajima K, Niisato N, Marunaka Y (2011). Genistein enhances the NGF-induced neurite outgrowth. Biomed Res.

[30] Nakajima K, Niisato N, Marunaka Y (2012). Enhancement of tubulin polymerization by Cl–induced blockade of intrinsic GTPase. Biochem Biophys Res Commun.

[31] Nakajima K, Niisato N, Marunaka Y (2012). Measurement of intracellular chloride concentration in differentiating PC12 cells. J Physiol Sci.

[32] Marunaka Y, Niisato N, Taruno A (2011). Regulation of epithelial sodium transport *via* epithelial Na^+^ channel. J Biomed Biotechnol.

[33] Niisato N, Taruno A, Marunaka Y (2007). Involvement of p38 MAPK in hypotonic stress-induced stimulation of beta- and gamma-ENaC expression in renal epithelium. Biochem Biophys Res Commun.

[34] Niisato N, Ohta M, Eaton DC (2012). Hypotonic stress upregulates beta- and gamma-ENaC expression through suppression of ERK by inducing MKP-1. Am J Physiol Renal Physiol.

[35] Marunaka Y, Niisato N, O'Brodovich H (1999). Regulation of an amiloride-sensitive Na^+^-permeable channel by a beta_2_-adrenergic agonist, cytosolic Ca^2+^ and Cl^−^ in fetal rat alveolar epithelium. J Physiol.

[36] Ohsawa R, Miyazaki H, Niisato N (2010). Intracellular chloride regulates cell proliferation through the activation of stress-activated protein kinases in MKN28 human gastric cancer cells. J Cell Physiol.

[37] Johansen T, Lamark T (2011). Selective autophagy mediated by autophagic adapter proteins. Autophagy.

[38] Weidberg H, Shvets E, Elazar Z (2011). Biogenesis and cargo selectivity of autophagosomes. Annu Rev Biochem.

[39] Zhang X, Tang N, Hadden TJ (2011). Akt, FoxO and regulation of apoptosis. Biochim Biophys Acta.

[40] Aharonovitz O, Kapus A, Szaszi K (2001). Modulation of Na^+^/H^+^ exchange activity by Cl. Am J Physiol Cell Physiol.

[41] Wang CZ, Yano H, Nagashima K (2000). The Na^+^-driven Cl^−^/HCO_3_^−^ exchanger. Cloning, tissue distribution, and functional characterization. J Biol Chem.

[42] Uchiyama Y (2001). Autophagic cell death and its execution by lysosomal cathepsins. Arch Histol Cytol.

[43] Hah YS, Noh HS, Ha JH (2012). Cathepsin D inhibits oxidative stress-induced cell death *via* activation of autophagy in cancer cells. Cancer Lett.

[44] Kaasik A, Rikk T, Piirsoo A (2005). Up-regulation of lysosomal cathepsin L and autophagy during neuronal death induced by reduced serum and potassium. Eur J Neurosci.

[45] Sapolsky AI, Altman RD, Woessner JF (1973). The action of cathepsin D in human articular cartilage on proteoglycans. J Clin Invest.

[46] Yonezawa S, Tanaka T, Miyauchi T (1987). Cathepsin E from rat neutrophils: its properties and possible relations to cathepsin D-like and cathepsin E-like acid proteinases. Arch Biochem Biophys.

[47] Jerala R, Zerovnik E, Kidric J (1998). pH-induced conformational transitions of the propeptide of human cathepsin L. A role for a molten globule state in zymogen activation. J Biol Chem.

[48] Turk B, Dolenc I, Lenarcic B (1999). Acidic pH as a physiological regulator of human cathepsin L activity. Eur J Biochem.

[49] Putney LK, Barber DL (2003). Na-H exchange-dependent increase in intracellular pH times G2/M entry and transition. J Biol Chem.

[50] Parks SK, Chiche J, Pouyssegur J (2013). Disrupting proton dynamics and energy metabolism for cancer therapy. Nat Rev Cancer.

[51] Bjorkoy G, Lamark T, Brech A (2005). p62/SQSTM1 forms protein aggregates degraded by autophagy and has a protective effect on huntingtin-induced cell death. J Cell Biol.

[52] Kabeya Y, Mizushima N, Ueno T (2000). LC3, a mammalian homologue of yeast Apg8p, is localized in autophagosome membranes after processing. EMBO J.

[53] Mizushima N, Yoshimori T (2007). How to interpret LC3 immunoblotting. Autophagy.

[54] Racoma IO, Meisen WH, Wang QE (2013). Thymoquinone inhibits autophagy and induces cathepsin-mediated, caspase-independent cell death in glioblastoma cells. PLoS ONE.

[55] Bartholomeusz C, Gonzalez-Angulo AM (2012). Targeting the PI3K signaling pathway in cancer therapy. Expert Opin Ther Targets.

